# Optimizing Immunohistochemical Surrogate Panels for 1p/19q Codeletion in IDH-Mutant Adult Diffuse Gliomas: Diagnostic Performance of Vimentin and H3K27me3

**DOI:** 10.3390/cancers18213588

**Published:** 2026-11-08

**Authors:** Eliza M. Löckli, Rachael S. Ninan, Krisztina A. Chalupa, Attila Mokánszki, Basil Habash, Ingrid Balogh, László Novák, Emanuel Gutema, Judit Bedekovics

**Affiliations:** 1 Department of Pathology, Faculty of Medicine, University of Debrecen, 4032 Debrecen, Hungarymokanszki.attila@med.unideb.hu (A.M.);; 2 Department of Oncology, Faculty of Medicine, University of Debrecen, 4032 Debrecen, Hungary; 3 Department of Neurosurgery, Faculty of Medicine, University of Debrecen, 4032 Debrecen, Hungary

**Keywords:** vimentin, H3K27me3, ATRX, astrocytoma, oligodendroglioma

## Abstract

Background: Differentiating IDH-mutant astrocytomas from IDH-mutant, 1p/19q-codeleted oligodendrogliomas can be challenging due to overlapping histology, particularly in small samples. We evaluated whether quantitative expression of vimentin and H3K27me3 can serve as reliable surrogate markers for 1p/19q-codeletion status. Methods: A cohort of 71 adult IDH-mutant diffuse gliomas (34 astrocytomas, 37 oligodendrogliomas) was analyzed. Marker expressions were evaluated together with key histopathological features. Results: Diagnostic performance was assessed using receiver operating characteristic (ROC) curves, multivariable logistic regression, and hierarchical modeling. Both H3K27me3 positivity (≥30% cut-off; 96.97% sensitivity, 88.89% specificity) and vimentin positive area (≥40% cut-off; 100% sensitivity, 89.19% specificity) were significantly higher in astrocytomas than in oligodendrogliomas (*p* < 0.0001). Classical oligodendroglial features correlated with lower expression of both markers, whereas gemistocytes correlated with higher expression. Individual AUCs were high for ATRX (0.933), H3K27me3 (0.928), and vimentin (0.944). In multivariable regression containing both markers, vimentin proved to be the dominant predictor (*p* = 0.008), rendering H3K27me3 redundant (*p* = 0.725). Hierarchical regression showed that adding vimentin or H3K27me3 to ATRX individually significantly improved performance (AUC = 0.994, *p* < 0.001). However, adding H3K27me3 to the ATRX + vimentin combination provided no significant improvement (*p* = 0.063). Conclusions: Vimentin loss and H3K27me3 loss are effective individual surrogate markers for 1p/19q-codeletion in adult IDH-mutant diffuse gliomas. However, multivariable and hierarchical regression modeling demonstrates that vimentin is the dominant predictor that renders H3K27me3 statistically redundant. A streamlined two-marker immunohistochemical panel combining ATRX and vimentin provides optimal diagnostic accuracy (AUC = 0.994) without requiring additional H3K27me3 testing, offering an accessible workflow for routine neuropathology.

## Simple Summary

Accurate subtyping of adult IDH-mutant diffuse gliomas—distinguishing astrocytomas from oligodendrogliomas—is essential for patient management, but histological overlaps may occur and molecular testing is required. Molecular analysis can be costly or unavailable on limited tissue samples. In this study, we evaluated the quantitative expression of vimentin and H3K27me3 as accessible surrogate markers for 1p/19q-codeletion status. Our findings demonstrate that while both markers perform well individually, combining vimentin with ATRX provides near-perfect diagnostic accuracy (AUC = 0.994), rendering additional H3K27me3 testing statistically redundant. By establishing a streamlined two-marker immunohistochemical panel, this study demonstrates a pragmatic, resource-sparing strategy for IDH-mutant diffuse glioma classification.

## 1. Introduction

Adult-type diffuse gliomas are classified by the 2021 World Health Organization (WHO) criteria using integrated diagnostics [1]. IDH-mutant oligodendrogliomas are molecularly defined by the 1p/19q-codeletion and characterized by “fried-egg” cellular morphology and “chicken-wire” vasculature [2,3,4]. Conversely, IDH-mutant astrocytomas harbor ATRX loss and *TP53* mutations, displaying a pleiomorphic, fibrillary matrix [5,6]. In routine pathology, however, clear-cut differentiation is frequently challenged by morphological mimicry between these tumors, especially in small or fragmented biopsy samples [1,2,3,4,5,6,7]. While gold-standard molecular assays like fluorescent in situ hybridization (FISH) or next-generation sequencing (NGS) provide definitive stratification, their high cost and long turnaround times underscore the demand for rapid and widely available immunohistochemical surrogate markers [1,8,9,10,11].

Histone H3p.K27me3 (H3K27me3) and vimentin are potential surrogate markers, as they reflect two distinct molecular pathways disrupted in these tumors: chromatin remodeling and intermediate filament expression. The driver *IDH* mutation produces the oncometabolite 2-hydroxyglutarate (2-HG), causing global epigenetic alterations [12,13]. Paradoxically, oligodendrogliomas often exhibit a distinct loss of the repressive H3K27me3 mark [14,15]. Concurrently, vimentin—a type III intermediate filament crucial for structural stability—is highly expressed in astrocytomas but characteristically absent or negligible in oligodendrogliomas [16,17,18,19,20].

This retrospective study aims to evaluate the quantitative expression of vimentin and H3K27me3 in a molecularly verified cohort of IDH-mutant adult diffuse gliomas and assess their correlation with specific histopathological features. Crucially, we aim to perform multivariate logistic regression analyses to determine whether the combined evaluation of vimentin and H3K27me3, assessed together with histopathological features and ATRX expression, can serve as a robust surrogate tool to effectively reproduce the results of 1p/19q-codeletion testing.

## 2. Methods

### 2.1. Patient Cohort

A total of 71 consecutive adult patients diagnosed and treated at the University of Debrecen were retrospectively identified and included in this analysis (IRB reference: IV/8465-3/2021/EKU; patient consent was waived due to the retrospective, anonymized nature). Inclusion criteria required confirmed *IDH* status and archived 1p/19q FISH results, while cases were excluded if patients were not treated at our institution or if tissue block exhaustion precluded comprehensive evaluation. Canonical *IDH1* mutation was confirmed by immunohistochemistry, and rare mutations by NGS. On the basis of 1p/19q-codeletion status, patients were classified into two groups: 37 cases (52.11%) were oligodendrogliomas with 1p/19q-codeletion and 34 cases (47.89%) were astrocytomas without 1p/19q-codeletion. The clinicopathologic characteristics of the entire cohort are presented in Table 1.

### 2.2. Histological Features

**Figure 1:**
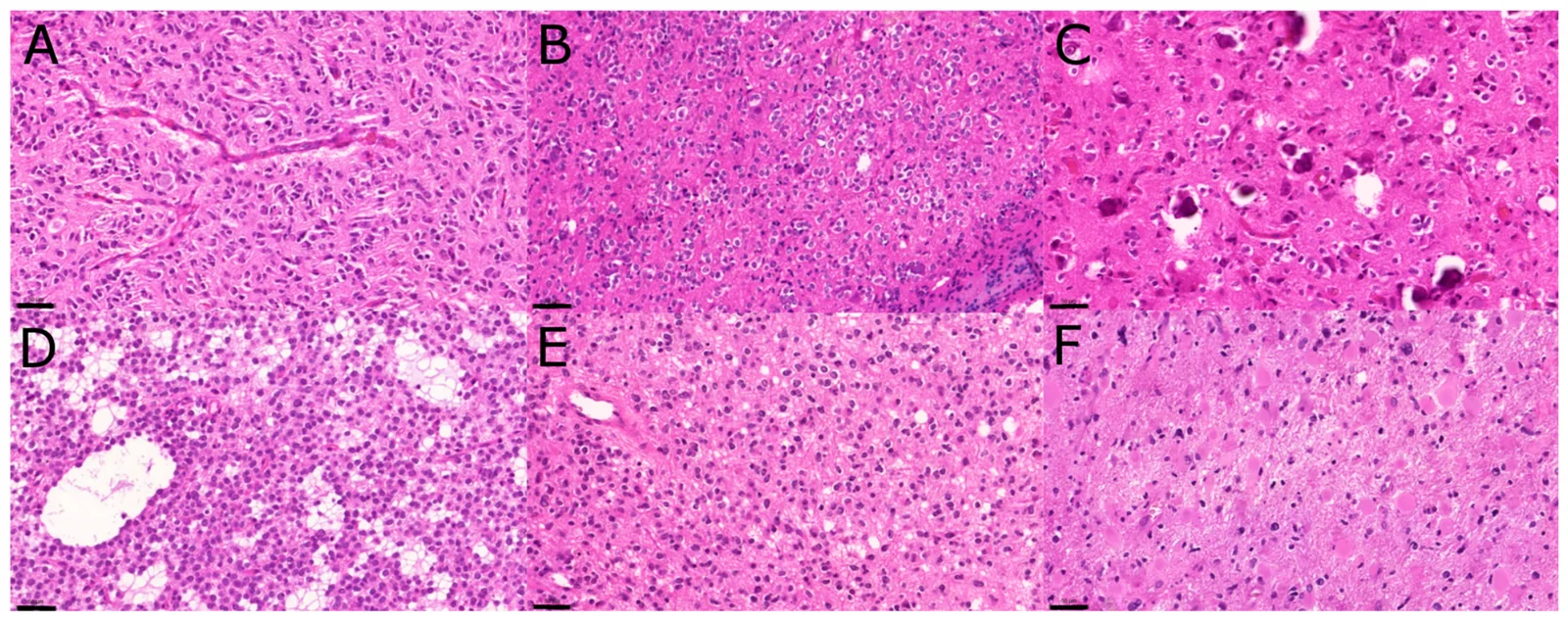
Histological features of IDH-mutant gliomas. The figure displays the various histological alterations that were assessed in all cases. (**A**) Branching vessel. (**B**) Perinuclear halo. (**C**) Calcification. (**D**) Microcystic areas. (**E**) Minigemistocytic areas. (**F**) Gemistocytic areas. (Scale bars indicate 50 µm).

Cases were examined for typical oligodendrogliomas or astrocytomas features. The presence of branching capillaries resembling chicken wire, perinuclear halo, calcifications, microcystic areas, minigemistocytic phenotype, and gemistocytic phenotype were individually evaluated and correlated with vimentin and H3K27me3 expression (Figure 1).

### 2.3. Immunohistochemistry

Four-micrometer-thick FFPE sections were used according to the manufacturer’s instructions for immunohistochemistry using the following primary antibodies: IDH1 p.R132H (mouse monoclonal, clone H09; BIOZOL, Eching, Germany; dilution 1:200; incubation: 32 min at RT), H3K27me3 (rabbit monoclonal, clone (EPR18607); Abcam, Cambridge, UK; dilution 1:50; incubation: 44 min at 37 °C), vimentin (mouse monoclonal, clone V9; Leica Biosystems, Nussloch, Germany; dilution 1:200; incubation: 40 min at 37 °C), ATRX (polyclonal; Merck Life Science, Darmstadt, Germany; dilution 1:1000; incubation: 36 min at 100 °C), and p53 (mouse monoclonal, clone DO-07; Dako, Agilent Technologies, Jena, Germany; dilution 1:700; incubation: 48 min at 37 °C). Antigen retrieval was performed using CC1 buffer (pH 8.5) at 100 °C for 36 (Vimentin and p53) or 48 (IDH1 p.R132H, H3K27me3, and ATRX) minutes. All immunohistochemical reactions were carried out on a VENTANA BenchMark ULTRA platform using the OPTIVIEW DAB detection kit (Ventana-Roche, Basel, Switzerland), DAB chromogen, and hematoxylin II counterstain.

### 2.4. Vimentin and H3k27me3 Expression

**Figure 2:**
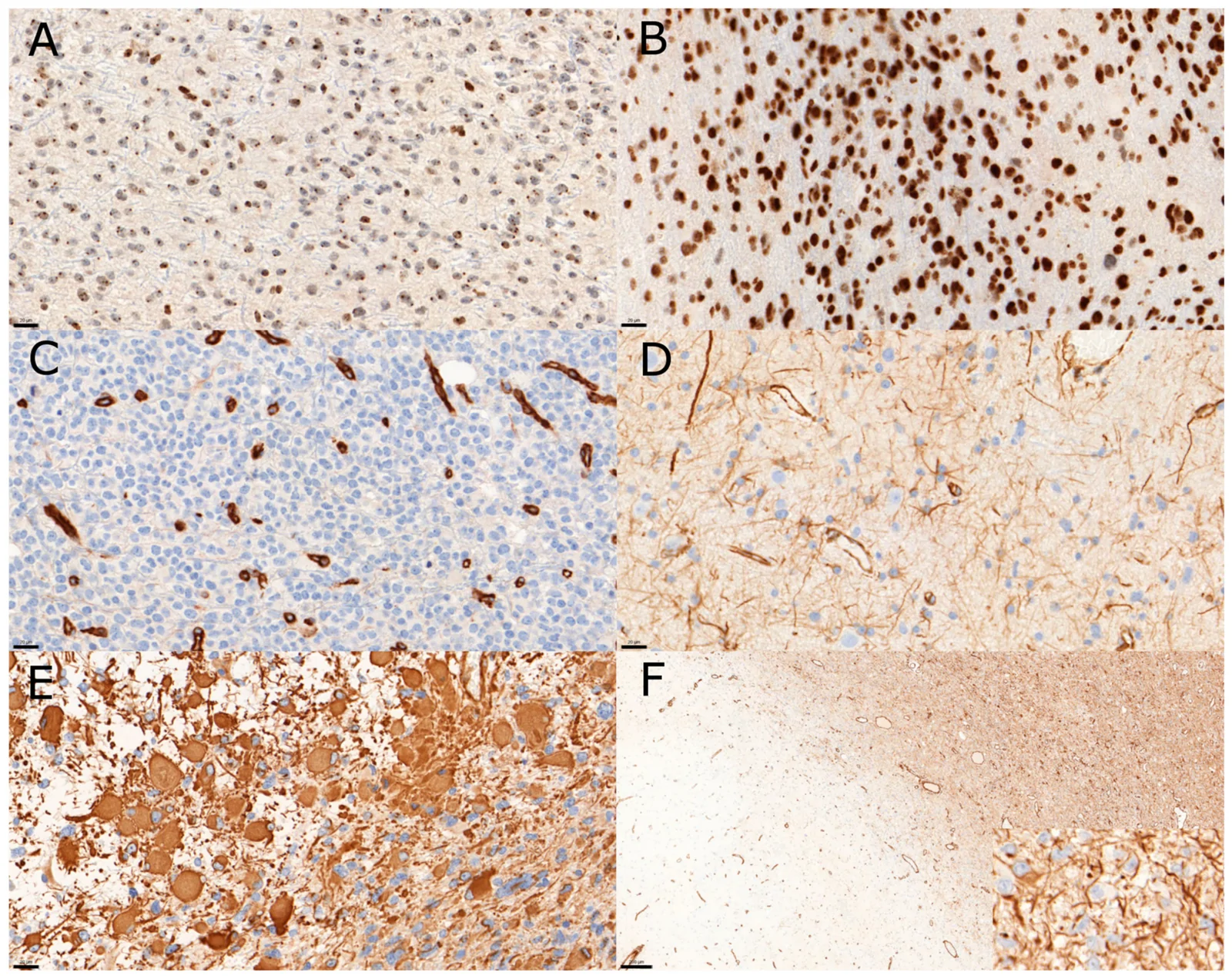
H3K27me3 and vimentin expression patterns in IDH-mutant gliomas. (**A**) Negative H3K27me3 staining in an oligodendroglioma sample from a female patient. Dot-like nuclear staining corresponding to the inactivated X chromosome is visible, which was excluded from the evaluation. (**B**) 100% H3K27me3 positivity in an astrocytoma from a male patient. (**C**) Representative image of a vimentin-negative oligodendroglioma sample, confirming the absence of vimentin expression in this oligodendroglioma tumor tissue. (**D**–**F**) Vimentin positive tumors diagnosed as astrocytoma. Positive vimentin expression displayed various patterns, such as predominantly fibrillary staining restricted to cell processes (**D**), predominantly cytoplasmic staining (**E**), and diffuse staining present in both components ((**F**), insert). (**F**) The infiltrative nature of the tumor is clearly highlighted by vimentin staining; the panel demonstrates the tumor edge. (Scale bars: (**A**–**E**) = 20 µm; (**F**) = 200 µm).

The level of H3K27me3 expression was evaluated as the percentage of tumor cells displaying nuclear staining of any intensity. The dot-like nuclear staining pattern resulting from inactivated X chromosome in female patients was excluded from scoring (Figure 2A,B) [21]. Due to the characteristic cellular morphology of diffuse glioma cells, which precludes reliable individual cell counting, vimentin expression was quantified semi-quantitatively as the percentage of positive tumor area regardless of staining intensity. Non-neoplastic intratumoral vessels, which strongly express vimentin, were visually identified and excluded from the scoring. To ensure consistency, all slides were evaluated jointly by two observers under a multi-head microscope to establish a consensus score. Vimentin positive cases displayed various staining patterns, including diffuse, fibrillary, and prominent cytoplasmic staining (Figure 2C–F). Stains were evaluated by two independent observers, and discrepant cases were resolved by consensus.

To rule out false-negative results, the staining quality of both ATRX and vimentin were evaluated in every case. A stain was considered valid only if retained nuclear expression (for ATRX) or strong cytoplasmic staining (for vimentin) was present in internal positive controls, such as vascular endothelial cells or non-neoplastic background cells. To prevent confirmation bias, immunohistochemical scoring was performed by pathologists who were blinded to the final 1p/19q FISH result.

H3K27me3 and vimentin expressions were correlated with tumor subtype (astrocytoma, IDH-mutant versus oligodendroglioma, IDH-mutant and 1p/19q-codeleted), histological features (branching capillaries resembling chicken wire, perinuclear halo, calcifications, microcystic areas, minigemistocytic phenotype, and gemistocytic phenotype), grade, and *IDH* mutation type.

Due to tissue exhaustion, H3K27me3 and vimentin staining was successfully evaluated in 69 and 70 cases, respectively, with 67 complete cases available for full multivariate modeling; all of the ATRX, H3K27me3, and vimentin stains were available.

### 2.5. Molecular Analysis

Fluorescence in situ hybridization (FISH) was performed on 5 μm thick sections of FFPE tissue blocks, using 1p36/1q25 and 19p13/19q13 deletion probes (MetaSystems, Altlussheim, Germany) separately, according to the manufacturer’s instructions. For FISH analysis, a 1p/19q co-deletion was defined as a signal ratio indicating deletion in more than 15% of evaluated non-overlapping tumor nuclei. These FISH probes evaluate targeted locus losses but do not directly distinguish whole-arm codeletions from partial chromosomal deletions. Rare IDH mutations were detected via NGS analysis in cases which were negative with IDH1 p.R132H immunohistochemistry. NGS was performed on FFPE samples using the RNA-based Archer FusionPlex Core Solid Tumor Panel (Archer DX, Boulder, CO, USA).

### 2.6. Statistical Analysis

Continuous variables were evaluated for normality, and non-parametric tests were performed using GraphPad Prism (v.8.0.1). Marker expression differences were assessed with Mann–Whitney U and Kruskal–Wallis tests, and categorical variables were compared using Fisher’s exact test. Correlations were evaluated using Spearman’s rank correlation. Diagnostic performance was determined via ROC curve analysis and Youden-index-based cut-offs. Single- and multivariable binary logistic regression models assessed independent predictive values. McFadden’s pseudo-R^2^, Akaike information criterion (AIC), and hierarchical models via the likelihood ratio test were calculated in Jamovi cloud (v.2.4) [22]. Two-tailed *p*-values < 0.05 were considered statistically significant.

## 3. Results

### 3.1. Clinicopathologic Characteristics

The cohort comprised 71 adults with diffuse gliomas: 34 (47.9%) IDH-mutant astrocytomas and 37 (52.1%) IDH-mutant, 1p/19q-codeleted oligodendrogliomas. The majority of patients in both groups were <55 year old, with a male dominance in the astrocytoma group (67.6%) compared to a more balanced gender distribution in oligodendrogliomas (48.6% male). The majority of tumors were located in the frontal lobe in both cohorts (astrocytoma 82.4%, oligodendroglioma 78.4%). Surgical resection was the primary specimen type in both groups (67.6% and 59.5%, respectively). The canonical *IDH1* p.R132H mutation was present in over 91% of cases in both groups. Consistent with their molecular definitions, ATRX loss (85.3%) and strong p53 expression (≥50%; 91.2%) were highly characteristic of astrocytomas, whereas oligodendrogliomas retained ATRX expression and typically showed low p53 expression. Most tumors were CNS WHO grade 2 in both the astrocytoma (67.6%) and oligodendroglioma (75.7%) groups. Oligodendrogliomas frequently exhibited classic histologic features, including branching vessels (75.7%), perinuclear halos (89.2%), calcifications (45.9%), and minigemistocytes (13.5%). Conversely, astrocytomas were characterized by a higher prevalence of gemistocytes (20.6%), while microcystic areas were commonly observed in both tumor subtypes (38.2% of astrocytomas and 45.9% of oligodendrogliomas; Table 1).

Marker expressions did not differ significantly between biopsy and resection specimens for either H3K27me3 (*p* = 0.3512) or vimentin (*p* = 0.7732), confirming their reliability in small diagnostic specimens.

### 3.2. Correlation of H3K27me3 Expression with Tumor Subtype and Histological Features

**Figure 3:**
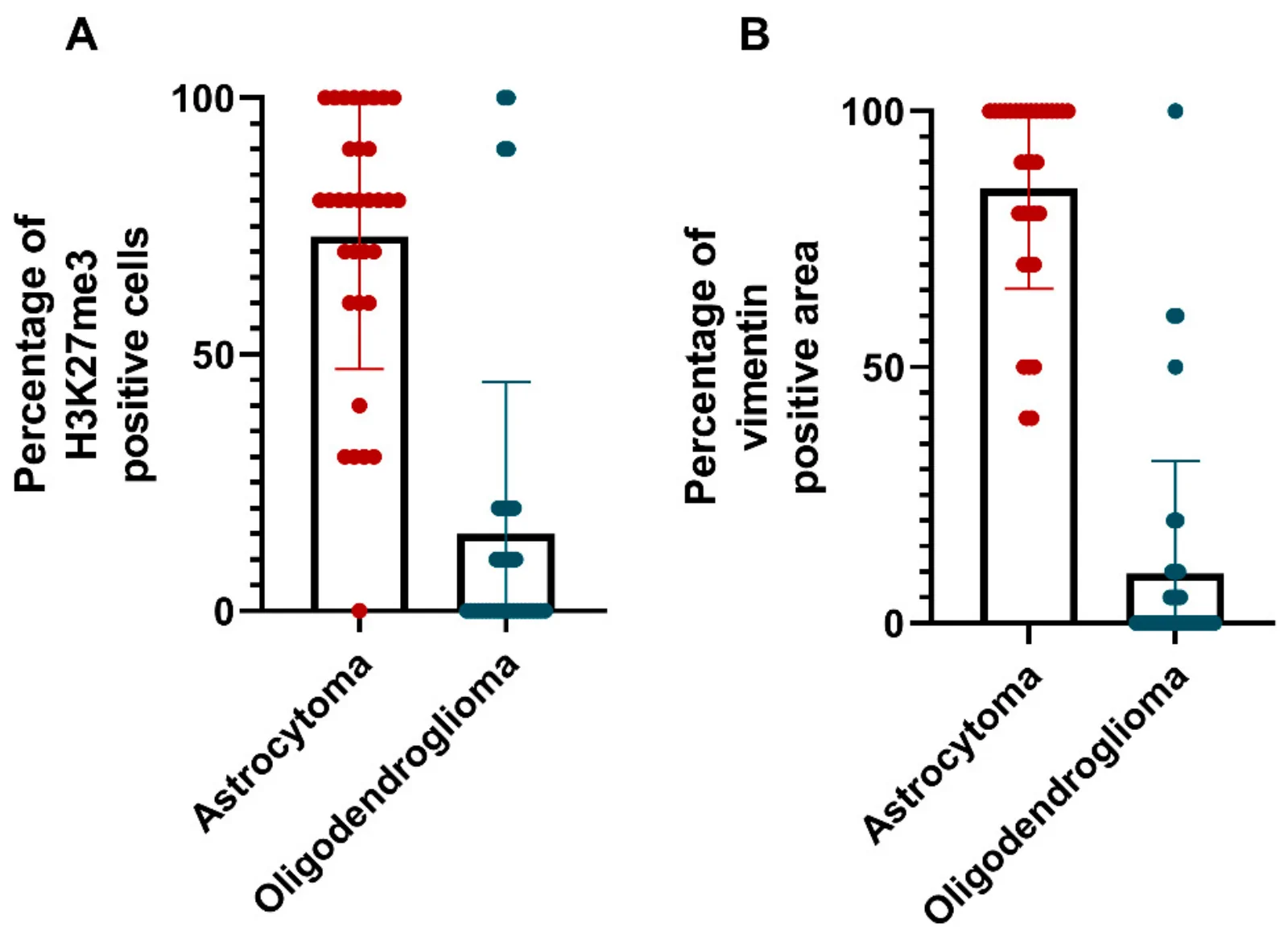
Expression levels of H3K27me3 and vimentin in IDH-mutant gliomas. (**A**) The percentage of H3K27me3-positive cells was significantly lower in IDH-mutant, 1p/19q-codeleted oligodendrogliomas compared to IDH-mutant astrocytomas (mean 73.03% vs. 15.00%, *p* < 0.0001, Mann–Whitney test). (**B**) The percentage of vimentin-positive area was significantly lower in IDH-mutant, 1p/19q-codeleted oligodendrogliomas compared to IDH-mutant astrocytomas (mean 84.85% vs. 9.73%, *p* < 0.0001, Mann–Whitney test).

The percentage of H3K27me3-positive tumor cells was significantly higher in IDH-mutant astrocytomas compared with IDH-mutant, 1p/19q-codeleted oligodendrogliomas (mean 73.03% vs. 15.00%, *p* < 0.0001, Mann–Whitney test; Figure 3A).

H3 K27me3 expression ranged from 0 to 100% across all cases. Sensitivity and specificity were calculated at 10% increments to determine the optimal cut-off point. Based on the Youden index (J = 0.8586), the optimal diagnostic cut-off was 30%, yielding a sensitivity of 96.97% and specificity of 88.89%. Consequently, cases with ≥30% expression were considered positive (Table 2).

Using this 30% threshold, 32/33 IDH-mutant astrocytomas and 4/36 IDH-mutant, 1p/19q-codeleted oligodendrogliomas were positive for H3K27me3 (*p* < 0.0001, Fisher’s exact test). The four positive oligodendroglioma cases showed 90–100% expression, and two were concurrently positive for vimentin. Both co-expressing cases showed grade 3 morphology, canonical IDH1 mutation, intact ATRX, and wild-type p53 expression.

The presence of branching vessels and perinuclear halos was significantly associated with a lower mean H3K27me3 expression (17.74% vs. 63.16%, *p* < 0.0001; and 18.06% vs. 69.70%, *p* < 0.0001, respectively). Similarly, calcification and minigemistocytes correlated with lower H3K27me3 expression (24.74% vs. 49.60%, *p* = 0.0326; and 6.00% vs. 45.63%, *p* = 0.0476, respectively). In contrast, gemistocytes were associated with higher expression (82.86% vs. 45.63%, *p* = 0.0023). Microcystic areas showed no significant difference (*p* = 0.2838). These results align with the classic oligodendroglial phenotype (Table 3). H3K27me3 expression did not differ across grades in astrocytomas (*p* = 0.9295, Kruskal–Wallis test) or oligodendrogliomas (*p* = 0.7906, Mann–Whitney test). The percentage of H3p.K27me3-positive tumor cells showed a significant correlation with the percentage of vimentin positive area (*p* < 0.0001, Spearman r = 0.6951).

### 3.3. Correlation of Vimentin Expression with Tumor Subtype and Histological Features

The percentage of vimentin-positive tumor cells was significantly higher in IDH-mutant astrocytomas compared with IDH-mutant, 1p/19q-codeleted oligodendrogliomas (mean 84.85% vs. 9.73%, *p* < 0.0001, Mann–Whitney test; Figure 3B). Based on the Youden index (J = 0.8919), the optimal diagnostic cut-off was 40%, yielding a sensitivity of 100% and specificity of 89.19%. Although 30% and 40% thresholds showed identical sensitivity and specificity, the 40% limit was selected as a more conservative, robust threshold for clinical use. Consequently, cases with ≥40% vimentin expression were considered positive (Table 2). Using the 40% cut-off threshold for vimentin-positive area, 33/33 IDH-mutant astrocytomas and 4/37 IDH-mutant, 1p/19q-codeleted oligodendrogliomas were positive for vimentin (*p* < 0.0001, Fisher’s exact test). The four positive oligodendroglioma cases showed 50–100% expression, and two were concurrently positive for H3K27me3 (as described above). Altogether, 6/37 (16.2%) IDH-mutant, 1p/19q-codeleted oligodendrogliomas showed either H3K27me3 or vimentin positivity, with co-expression present in only 2 cases (2/37, 5.41%). Both of these outliers were CNS WHO grade 3 tumors lacking classical perinuclear halos. Both cases demonstrated retained ATRX expression, wild-type p53 status, and clear 1p/19q codeletion patterns confirmed by FISH. Topographically, one tumor was located in the frontal lobe and the other in the insular area. Vimentin expression showed a histological pattern similar to H3K27me3. Branching vessels and perinuclear halos were significantly associated with reduced vimentin expression (14.35% vs. 69.62%, *p* < 0.0001; and 12.70% vs. 81.52%, *p* < 0.0001, respectively). Calcification also correlated with lower expression (15.71% vs. 52.32%, *p* = 0.0134). In contrast, gemistocytic features were associated with higher expression (91.67% vs. 40.78%, *p* = 0.0036). Microcystic areas and minigemistocytic morphology showed no statistically significant association (*p* = 0.2538 and *p* = 0.0624, respectively; Table 3). Vimentin expression did not correlate with WHO grade in IDH-mutant astrocytomas (*p* = 0.2344, Kruskal–Wallis test) or IDH-mutant, 1p/19q-codeleted oligodendrogliomas (*p* = 0.4169, Mann–Whitney test).

### 3.4. Multivariate Analysis of H3K27me3, Vimentin Expression, and Histopathological Features

**Figure 4:**
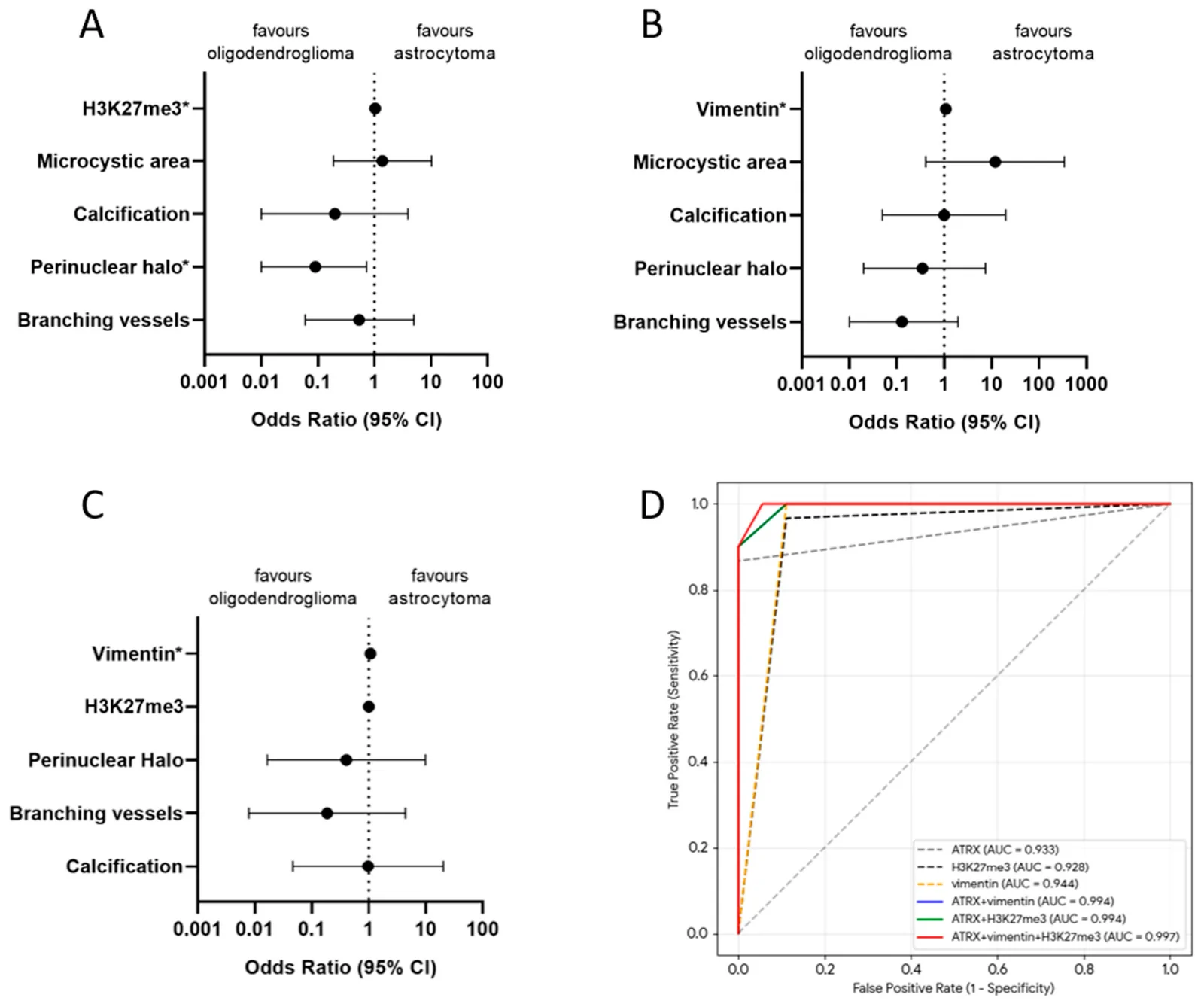
Forest plots of the multivariate logistic regression models. Data points show the odds ratios (OR) with 95% confidence intervals (CI). The vertical dashed line marks the line of no effect (OR = 1.0); values to the right favor IDH-mutant astrocytoma, and values to the left favor IDH-mutant, 1p/19q-codeleted oligodendroglioma. Asterisk indicates statistically significant independent predictors. Histological features with extreme ORs and disproportionately wide confidence intervals, such as gemistocytic/minigemistocytic areas and microcystic features, were excluded to prevent visual distortion of plot scale. (**A**) H3K27me3-only model: H3K27me3 is a significant independent predictor (*p* = 0.007). (**B**) Vimentin-only model: vimentin shows high predictive value (*p* = 0.003). (**C**): Combined model: both markers evaluated together. Vimentin remains significant (*p* = 0.008), while H3K27me3 loses significance (*p* = 0.725). Both H3K27me3 and vimentin show a narrow confidence interval, underscoring the robustness of the predictive value. (**D**) Comparative ROC curves for individual immunohistochemical markers and combined multi-marker panels. Dashed lines represent individual markers (ATRX, H3K27me3, vimentin), whereas solid colored lines denote multi-marker panels generated via logistic regression models (ATRX + vimentin, ATRX + H3K27me3, and the three-marker panel) (N = 67). Area under the curve (AUC) values: ATRX = 0.933; H3K27me3 = 0.928; vimentin = 0.944; ATRX + vimentin = 0.994; ATRX + H3K27me3 = 0.994; three-marker panel = 0.997. Note that the ROC curves for the two-marker panels ATRX + vimentin (blue) and ATRX + H3K27me3 (green) completely overlap due to identical AUC performance (AUC = 0.994).

To comprehensively evaluate the diagnostic performance and interplay of H3K27me3 and vimentin markers, separate and combined multivariable logistic regression models were performed together with histopathological features (Figure 4A–C).

In the first model, where H3K27me3 was analyzed separately, quantitative H3K27me3 expression was a statistically significant independent predictor of the final molecular diagnosis (*p* = 0.007, OR = 1.035, 95% CI: 1.010–1.061). In this analysis, perinuclear halo was also a significant predictor (*p* = 0.024, OR = 0.086, 95% CI: 0.010–0.725). The H3K27me3-only model demonstrated a strong overall fit, explaining 65.1% of the diagnostic variance (McFadden R^2^ = 0.651).

In the second model, vimentin expression was analyzed separately and showed an even stronger independent predictive value (*p* = 0.003, OR = 1.084, 95% CI: 1.028–1.143). The vimentin-only model provided a superior overall fit (McFadden R^2^ = 0.795).

Finally, when both biomarkers were integrated into a combined multivariable analysis, the overall model fit remained highly robust (McFadden R^2^ = 0.792). However, vimentin expression emerged as the dominant predictor (*p* = 0.008), rendering H3K27me3 statistically non-significant (*p* = 0.725). These findings indicate that while both biomarkers have independent diagnostic value separately, vimentin is the dominant predictor when analyzed simultaneously, making H3K27me3 statistically redundant.

### 3.5. ROC Analysis, Hierarchical Logistic Regression, and Biomarker Combination Analysis

All individual immunohistochemical markers demonstrated high diagnostic performance, with AUC values of 0.933 for ATRX, 0.928 for H3K27me3, and 0.944 for vimentin (Figure 4D). Combining markers into multivariable logistic regression models substantially improved diagnostic accuracy. Both two-marker combinations (ATRX + vimentin and ATRX + H3K27me3) achieved an AUC of 0.994, while the three-marker panel (ATRX + vimentin + H3K27me3) achieved an AUC of 0.997.

To further evaluate whether adding vimentin or H3K27me3 provides additional diagnostic value over ATRX alone, hierarchical binary logistic regression models were compared using the likelihood ratio test. ATRX alone demonstrated strong baseline diagnostic utility (McFadden R^2^ = 0.719, AIC = 30.0). Individually adding either vimentin or H3K27me3 to ATRX yielded identical, highly significant performance gains (χ^2^ = 14.9, *p* < 0.001, McFadden R^2^ = 0.880, AIC = 17.1 for both models). However, adding H3K27me3 to the combined ATRX + vimentin model provided no statistically significant improvement (χ^2^ = 3.45, *p* = 0.063; McFadden R^2^ = 0.917).

Given the strong independent predictive value of vimentin, the ATRX + vimentin panel represents the optimal diagnostic combination, beyond which adding H3K27me3 offers no significant independent gain.

## 4. Discussion

The WHO Classification of Tumors of the Central Nervous System emphasizes an integrated phenotypic and molecular diagnostic approach for diffuse gliomas. The presence of 1p/19q-codeletion in IDH-mutant gliomas is the defining molecular event for the diagnosis of oligodendroglioma, distinguishing it from IDH-mutant astrocytoma. Although molecular techniques such as FISH remain the gold standard for detecting 1p/19q-codeletion, there is a need for reliable, cost-effective surrogate immunohistochemical markers [1,8,9,23].

Vimentin is a type III intermediate filament protein that plays a fundamental role in maintaining cellular cytoarchitecture, mechanical integrity, cellular motility, and signal transduction [16,17,18,19,20]. In the developing central nervous system, vimentin is prominently expressed in radial glial cells, progenitor cells, and immature astrocytes [24,25,26]. As neurodevelopment progresses, mature oligodendrocytes typically downregulate vimentin expression [17,18]. This physiological lineage-specific difference provides a strong biological rationale for using vimentin expression patterns to distinguish astrocytic tumors from oligodendroglial lineage neoplasms [19,20].

Conversely, the lack of nuclear H3K27me3 in 1p/19q-codeleted oligodendrogliomas presents an epigenetic paradox, as IDH mutation-induced 2-HG accumulation typically inhibits histone demethylases [12,13,14,15,27]. The global loss of H3K27me3 in oligodendrogliomas is hypothesized to stem from chromatin remodeling secondary to structural genomic alterations, such as 1p/19q-codeletion involving CIC and FUBP1 [28]. Although the exact molecular interplay between 1p/19q loss and H3K27 trimethylation dynamics remains to be fully elucidated, this loss serves as a robust hallmark distinguishing oligodendrogliomas from IDH-mutant astrocytomas, which consistently retain H3K27me3 [14,15].

Mutations in CIC and FUBP1 represent hallmark genetic alterations in 1p/19q-codeleted oligodendrogliomas, and immunohistochemical detection of their protein expression offers a further surrogate approach for 1p/19q status. Incorporating CIC and FUBP1 IHC provides potential complementary diagnostic tools alongside established markers in comprehensive neuropathological analysis [29,30].

We evaluated the diagnostic utility of H3K27me3 and vimentin loss as surrogate IHC markers for 1p/19q-codeletion. The loss of both markers strongly correlated with oligodendroglioma vs. astrocytoma (*p* < 0.0001), aligning with previous reports [14,15,19,20,31,32].

While relying on a single marker can lead to diagnostic pitfalls, marker combinations significantly enhance accuracy. Individual ROC analysis demonstrated high performance for ATRX (AUC = 0.933), H3K27me3 (AUC = 0.928), and vimentin (AUC = 0.944). In hierarchical modeling, adding either vimentin or H3K27me3 to ATRX improved diagnostic performance significantly (AUC = 0.994, *p* < 0.001). Although earlier studies evaluated these markers separately, our study is the first to model their combined diagnostic value alongside ATRX in a multivariable framework.

Notably, while recent studies—such as the three-marker panel proposed by Wadekar et al. (2025) [32]—advocate for expanding diagnostic panels by combining multiple biomarkers (including Hip1R, vimentin, and H3K27me3), our multivariable analysis demonstrates that larger panels do not necessarily yield superior diagnostic utility. In our cohort, vimentin emerged as the dominant independent predictor (*p* = 0.008), rendering H3K27me3 statistically non-significant (*p* = 0.725). Furthermore, hierarchical modeling confirmed that adding H3K27me3 to the combined ATRX + vimentin panel provided no statistically significant diagnostic gain (*p* = 0.063). Consequently, an optimized two-marker panel (ATRX + vimentin) is fully sufficient to achieve optimal diagnostic accuracy (AUC = 0.994). Additionally, as demonstrated in our multivariable logistic regression analysis (Figure 4A–C), individual histological features lost independent statistical significance when evaluated alongside molecular surrogate markers. This suggests that classical histological features represent collinear morphological correlates of the specific tumor subtype rather than independent diagnostic predictors.

Neither H3K27me3 nor vimentin expression showed significant variation across CNS WHO tumor grades 2 and 3 within the respective astrocytoma and oligodendroglioma subgroups (*p* > 0.05), though these findings should be interpreted with caution given the relatively low representation of grade 3 cases in our cohort.

Study limitations include the relatively small sample size (n = 71) and single-center nature. The proposed cut-off values were derived and evaluated within our single-center internal cohort. While the two-marker model achieved an excellent diagnostic accuracy (AUC = 0.994), due to modest cohort size and the single-center nature of the study, there is a potential risk of internal model overoptimism. Therefore, validation in larger, independent external cohorts is necessary before widespread clinical adoption. Additionally, due to the predominance of WHO grade 2 tumors, our study had limited statistical power to evaluate marker expression variations across higher histological grades. While immunohistochemical evaluation of CIC and FUBP1 represents a further relevant strategy for characterizing 1p/19q-codeleted oligodendrogliomas, these markers were not included in our present staining panel. Our study focused specifically on evaluating ATRX, vimentin, and H3K27me3 to test a minimal, highly accessible, and cost-effective initial screening combination. However, integrating CIC and FUBP1 into future multi-center validation studies might be highly valuable to refine combined diagnostic algorithms.

Furthermore, our cohort was restricted to adult IDH-mutant diffuse gliomas. Importantly, these immunohistochemical alterations—particularly H3K27me3 loss—are not entirely specific to oligodendrogliomas, as trimethylation loss also occurs in other CNS neoplasms [33]. Therefore, these cutoff values and staining patterns must be interpreted strictly within the context of adult IDH-mutant tumors.

While formal cost-effectiveness analyses were not performed in this study, implementing this two-marker IHC panel offers a pragmatic and economically favorable screening workflow. Compared to routine primary molecular testing, initial IHC screening reduces direct laboratory reagent expenses and turnaround time, reserving molecular testing for ambiguous cases.

## 5. Conclusions

In conclusion, our findings demonstrate that vimentin loss and H3K27me3 loss are highly effective surrogate markers for 1p/19q-codeletion in adult IDH-mutant diffuse gliomas. Crucially, combining ATRX with vimentin alone offers optimal diagnostic accuracy without requiring redundant H3K27me3 testing, providing a streamlined and cost-conscious approach for routine neuropathology workflows.

## Figures and Tables

**Table 1:** Clinicopathologic characteristics of the entire cohort of adult diffuse gliomas (n = 71) stratified by the integrated diagnosis.

Clinicopathologic Characteristics (n = 71)		Astrocytoma, IDH Mutant (n = 34)		Oligodendroglioma, IDH-Mutant and 1p/19q-Codeleted (n = 37)	
		N	(%)	N	(%)
Age	<55	29	85.3%	28	75.7%
	≥55	5	14.7%	9	24.3%
Sex	Male	23	67.6%	18	48.6%
	Female	11	32.4%	19	51.3%
Tumor location by dominant lobe involvement	Frontal	28	82.4%	29	78.4%
	Parietal	3	8.8%	4	10.8%
	Temporal	2	5.9%	4	10.8%
	Occipital	1	2.9%	0	-
Specimen type	Biopsy	11	32.4%	15	40.5%
	Resection	23	67.6%	22	59.5%
*IDH* status	IDH1 p.R132H	31	91.2%	34	91.9%
	other	3	8.8%	3	8.1%
ATRX status via IHC *	ATRX loss	29	85.3%	0	-
	ATRX intact	4	11.8%	37	100%
p53 status via IHC	<50% expression	3	8.8%	31	83.8%
	≥50% expression	31	91.2%	6	16.2%
CNS WHO grade	grade 2	23	67.6%	28	75.7%
	grade 3	4	11.8%	9	24.3%
	grade 4	7	20.6%	NA	-
Histologic feature present	branching vessels	4	11.8%	28	75.7%
	perinuclear halo	4 ^#^	11.8%	33	89.2%
	calcification	3	8.8%	17	45.9%
	microcystic areas	13	38.2%	17	45.9%
	minigemistocytic	0	-	5	13.5%
	gemistocytic	7	20.6%	0	-

* In one case of IDH-mutant astrocytoma, ATRX expression was not available. ^#^ In three out of four IDH-mutant astrocytoma cases, perinuclear halo was a focal feature. NA: not applicable.

**Table 2:** Sensitivity, specificity, and Youden index of H3K27me3 and vimentin expression to determine the optimal cut-off value. Based on the evaluation of the Youden index, the optimal diagnostic cut-off value was identified at 30% for H3K27me3 and at 40% for vimentin expression.

	H3K27me3 Expression			Vimentin Expression		
Cut-Off Value (Greater than or Equal to)	Sensitivity	Specificity	Youden Index	Sensitivity	Specificity	Youden Index
10%	0.9697	0.5833	0.5530	1.000	0.7568	0.7568
20%	0.9697	0.7500	0.7197	1.000	0.8378	0.8378
30%	0.9697	0.8889	0.8586	1.000	0.8919	0.8919
40%	0.8485	0.8889	0.7374	1.000	0.8919	0.8919
50%	0.8182	0.8889	0.7071	0.9394	0.8919	0.8313
60%	0.8182	0.8889	0.7071	0.8485	0.9189	0.7674
70%	0.7273	0.8889	0.6162	0.8485	0.9730	0.8215
80%	0.6061	0.8889	0.4950	0.7576	0.9730	0.7306
90%	0.3333	0.8889	0.2222	0.6061	0.9730	0.5791
100%	0.2424	0.9444	0.1868	0.4848	0.9730	0.4578

**Table 3:** Correlation of H3K27me3 and vimentin expression with histological features.

Histological Feature		H3K27me3 Expression		Vimentin Expression	
		Mean Expression (% of Tumor Cells)	*p*-Value *	Mean Expression (% of Area)	*p*-Value *
branching vessels	present	17.74	<0.0001	14.35	<0.0001
	absent	63.16		69.62	
perinuclear halo	present	18.06	<0.0001	12.70	<0.0001
	absent	69.70		81.52	
calcification	present	24.74	0.0326	15.71	0.0134
	absent	49.60		52.32	
microcystic areas	present	37.59	0.2838	37.67	0.2538
	absent	46.50		50.75	
minigemistocytic	present	6.000	0.0476	4.000	0.0624
	absent	45.63		48.31	
gemistocytic	present	82.86	0.0023	91.67	0.0036
	absent	38.23		40.78	

* Mann–Whitney test.

## Data Availability

The data that support the findings of this study are available from the corresponding author upon reasonable request.

## Abbreviations

AUC	area under the curve
CI	confidence interval
CNS	central nervous system
FFPE	formalin-fixed, paraffin-embedded
FISH	fluorescence in situ hybridization
H3K27me3	histone H3 lysine 27 trimethylation
IDH	isocitrate dehydrogenase
IHC	immunohistochemistry
OR	odds ratio
NGS	next-generation sequencing
WHO	World Health Organization
2-HG	2-hydroxyglutarate
